# Knowing equity, doing equity: healthcare cultures and why reforms stall in everyday care

**DOI:** 10.3389/fpubh.2026.1817621

**Published:** 2026-05-08

**Authors:** Ángel Martínez-Hernáez, Francisco Ortega, Ivo Quaranta, Helena Hansen

**Affiliations:** 1 Medical Anthropology Research Center, Universitat Rovira i Virgili, Tarragona, Spain; 2 Catalan Institution for Research and Advanced Studies (ICREA), Barcelona, Spain; 3 Department of History and Cultures, Alma-Mater Studiorum-Università di Bologna, Bologna, Italy; 4 Department of Psychiatry and Biobehavioral Sciences, David Geffen School of Medicine, Jane and Terry Semel Institute for Neuroscience and Human Behavior, University of California Los Angeles, Los Angeles, CA, United States

**Keywords:** health disparities, health equity, healthcare cultures, institutional reflexivity, public health evaluation, social determinants of health, structural inequity, transformative care

## Abstract

Health systems have multiplied equity-oriented frameworks over recent decades, yet inequities remain embedded in routine care. We argue that this persistent gap reflects a structural blind spot: culture is typically treated as an external attribute of patients or communities rather than as a constitutive dimension of healthcare itself. Reframing healthcare as culturally organised reveals how classification, knowledge validation, and criteria of efficiency shape what problems become visible, whose accounts are legitimised, and which responses are deemed appropriate. We introduce an analytical framework and typology of non-reflexive, adaptive, and transformative healthcare cultures to clarify why equity reforms often underperform and how institutional reflexivity can become operational. The framework includes a set of operational indicators, organised by mechanism and level of analysis, that can be used to evaluate healthcare cultures empirically and support institutional reform across settings. For public health, the implication is direct: evaluation and governance must move upstream, beyond downstream outcomes, to examine the cultural infrastructures that organise routine decision making. Equity becomes not an added objective, but a property of how care is structured, enacted, and assessed.

## Introduction: the equity paradox

Health systems across the world face a persistent paradox. Despite decades of reforms and major investments, inequities remain embedded in everyday care. Over the same period, equity-oriented frameworks have multiplied, including the social determinants of health (1), cultural competence (2), structural competency (3–5), rights-based and people-centred approaches (6), community participation (7), and shared decision making (6). These frameworks have expanded how health systems conceptualise equity, yet their impact on practice remains limited. They have rarely altered how biomedical systems organise care. Many are implemented as add-ons that leave the core organisation of care unchanged. This fragmentation reflects a persistent gap between institutional discourse and everyday practice. Equity agendas often circulate as formal commitments but are not internalised as part of clinical reasoning or what health systems enact as “good care” (8). When these perspectives lack a shared architecture linking analysis, organisational design, and evaluation across the system, equity remains confined to policy documents and fails to become an organising principle of routine decision making. The problem, therefore, lies in the limited capacity of health systems to translate equity-oriented frameworks into routine organisational practice.

This gap has a specific source. It arises from the tendency to treat culture as an external context rather than as a constitutive element of care in biomedical practice. Culture is typically located in patients, communities, and “belief systems,” to be managed through communication strategies, competence checklists, or tailored interventions (2, 9). As a result, the cultures of services and policies themselves remain weakly theorised. The decoupling between institutional declarations and routine practice is therefore structural, embedded in how systems classify problems, validate knowledge, and define appropriate action.

Understanding why culture matters therefore requires a shift in how healthcare is conceptualised. Healthcare extends beyond a technical response to illness. It operates as a culturally organised habitus (10) through which health systems define what counts as a problem, who is recognised as a credible narrator, which risks deserve attention, and what kinds of suffering are recognised as legitimate (9–13). These processes cannot be reduced to individual attitudes. They are institutionally sedimented across medical curricula, clinical protocols, documentation standards, and performance regimes. Seen in this way, culture forms part of the institutional machinery through which decisions are produced and justified.

Three bodies of scholarship have engaged directly with the cultural dimensions of care, each from a different angle. Medical anthropology and allied fields have shown that biomedicine is irreducibly cultural, shaped by classificatory regimes, moral orders, and institutional power (9–13). This work has been effective in analysing classificatory regimes and institutional power in biomedicine; however, it has rarely been translated into tools for organisational redesign. In parallel, organisational and health services research has mobilised culture through concepts such as safety culture, quality culture, teamwork, and organisational climate (14–17). These approaches support measurement and performance improvement, but conceptualise culture as a narrow managerial variable, detached from how priorities and legitimacy are produced. Care ethics and feminist scholarship, for their part, have reframed care around interdependence, relationality, and justice (18, 19). Yet these insights remain marginal to health system governance and are often confined to informal or community care, leaving the cultural organisation of professional care largely unexamined (20). Together, these traditions provide important insights, yet they remain weakly integrated at the level of public health evaluation.

Together, these limitations explain why equity reforms repeatedly underperform. The problem lies in the siloing of existing approaches from the cultural organisation of routine care. This perspective therefore starts from a different premise: care is not merely shaped by culture; care is culturally organised. The categories, routines, metrics, and decision logics of health services are themselves cultural formations that structure how inequity is recognised, translated into cases, and acted upon (11, 12). This perspective contributes to the literature in three ways: by reconceptualising culture as an institutional infrastructure of healthcare; by introducing three analytical mechanisms (classification, knowledge validation, and criteria of efficiency) to examine how inequities are produced in routine care; and by proposing a typology of healthcare cultures (non-reflexive, adaptive, and transformative) that can support comparative analysis, public health evaluation, and organisational reform.

## Rethinking healthcare cultures

Healthcare cultures can be defined as structured configurations of knowledge, values, and power that shape, through the mechanisms of classification, knowledge validation, and criteria of efficiency, how health systems produce cases, authorise interventions, and allocate legitimacy. They are not reducible to individual beliefs. They are patterned and institutionalised processes enacted across levels, in encounters, in service organisation, and in governance (21). They encompass what is said and enacted, as well as what is coded, recorded, audited, and rewarded through routine institutional procedures. In other words, they describe the operating logics that make some inequities legible and actionable and others invisible. These configurations can be systematically analysed as institutional processes.

This definition shifts how the paradox is understood. When culture is treated as mere context, equity reform is imagined as something external to care, for example through training modules, checklists, rights statements, or participation forums. When culture is treated as constitutive, equity becomes an internal property of care organisation. It is produced upstream, before outcomes are measured, through the cultural mechanisms that shape recognition, voice, and legitimacy in routine practice. Healthcare therefore operates through cultural operations (classification, knowledge validation, and criteria of efficiency) that organise what counts as “good care.” These mechanisms constitute an analytical framework through which healthcare cultures can be examined across settings, organisations, and policy contexts.

This framework can be illustrated through routine clinical scenarios. Consider a hypothetical but typical case across urban systems such as Barcelona, Oslo, Los Angeles, Bologna, or Rio de Janeiro. A migrant patient experiencing poverty and racism presents with distress, fatigue, and distrust. In the electronic record, the case is stabilised as “depression” and “non-compliance.” The system provides no place to register structural conditions such as racism, labour exploitation, or housing precarity. This absence is consequential. It translates social suffering into an individual deficit and reshapes what the system can recognise, justify, and act upon (5, 22).

Persistent racial disparities in maternal mortality in the United States illustrate this dynamic. Despite sustained policy attention and equity-oriented initiatives, Black women continue to experience maternal mortality rates several times higher than those of White women, reflecting deeply ingrained structural inequities (23, 24). These disparities have been linked to the enduring effects of anti-Black racism within clinical practice, medical training, and health delivery systems, shaping risk classification, care trajectories, and institutional response patterns in ways that remain largely invisible to conventional evaluation frameworks (23). As a result, reforms centred on implicit bias training and awareness campaigns coexist with classification systems and performance regimes that leave the underlying cultural operations of care largely unchanged. In both cases, classification, knowledge validation, and criteria of efficiency operate in ways that obscure structural exposure and limit institutional response.

A transformative configuration of care generates a different case logic: structural exposure is recorded as decision-relevant, responsibility is distributed across clinical and social response, and success is assessed by reduced exposure and improved function, alongside symptom change or adherence. When biomedical systems recognise this suffering as socially produced, and within their purview to treat, they have begun to address housing as a health intervention, collaborate with labour and racial equity organisations, and advocate for racial equity as core health issues within a “health in all policies” approach. Under this configuration, health services act as institutional partners within a broader integrated health and social care system, integrating clinical decision-making with social service coordination and intersectoral governance rather than displacing these functions onto clinicians alone.

Healthcare cultures can be made empirically tractable through three interrelated mechanisms that function as analytical dimensions of the framework: what problems become actionable, whose knowledge is recognised as valid, and what counts as an appropriate response in practice. This framework operates across three analytical levels: the micro level of clinical encounters, the meso level of organisational routines, and the macro level of policy and governance structures.

- *Classification*: clinical and institutional categorisation defines what counts as illness, need, and legitimate intervention. When “non-compliance” replaces poverty or racism, the category structures what the system can do. Classification systems translate situations into actionable cases.
- *Knowledge validation*: institutions privilege certain forms of evidence while discounting others. Clinical expertise and managerial data typically carry institutional authority, while experiential and community knowledge are often treated as supplementary. These hierarchies are embedded in documentation standards, encounter formats, and accountability structures that determine which accounts are recognised as decision-relevant.
- *Criteria of efficiency*: institutional values such as efficiency and risk management become embedded in routine practice through targets, metrics, and performance regimes (16, 17). These criteria define which forms of care are prioritised, routinised, or sidelined in everyday practice.

These mechanisms help explain why equity frameworks often coexist without integration. Structural competency, rights-based approaches, shared decision making, and participatory frameworks each engage with one dimension of the problem (awareness, entitlement, voice, or inclusion) without reconfiguring the institutional logic that governs all three mechanisms simultaneously. These approaches are not flawed in themselves. Their limits arise when they are implemented in isolation, without reconfiguring the institutional logic that governs classification, validation, and efficiency criteria. Each addresses a symptom; none revises the underlying cultural infrastructure through which care is organised. Without that reconfiguration, reforms tend to be additive rather than transformative.

Different configurations of these mechanisms produce distinct types of healthcare cultures. These types differ in how clinical and institutional categorisation, knowledge validation, and criteria of efficiency are configured and made reflexive in practice.

- *Non-reflexive healthcare* cultures sustain inequity through routinised care habitus (10) in which routine classification erases social exposure and institutional responses recode vulnerability as individual deficit. Patients’ needs are defined by what services are equipped to offer, while clinical practice reproduces the implicit cultural assumptions embedded in institutional routines. Because categorisation, knowledge validation, and criteria of efficiency remain unquestioned, exclusion persists under conditions of apparent clinical coherence. Structural exposures are not recorded or are reframed as non-adherence or risk behaviour, with the result that social injustices are not merely obscured but actively reproduced by systems of care.
- *Adaptive healthcare cultures* adjust partially in response to critique or crisis. They may revise protocols, add social questions, or open participatory spaces, but the underlying configuration of categorisation, knowledge validation, and criteria of efficiency remains largely unchanged. Equity is incorporated as a secondary layer rather than as an organising principle. Social determinants may be documented, yet they remain weakly connected to decision authority or resource allocation.
- *Transformative healthcare cultures* enact reflexive habitus capable of revising how problems are categorised, whose knowledge is validated, and how efficiency is defined. They include participation, redistribute decision authority, and revise infrastructures so that structural vulnerability becomes legible and actionable. For example, this involves co-designing intake forms with community health workers to capture social determinants, creating institutional spaces where patient narratives carry weight in case reviews, or revising performance metrics to reward relational continuity and reductions in inequitable outcomes alongside throughput. The criterion is practical: the system revises its own cultural operations rather than simply adding new programmes. A minimal indicator is whether structural exposure changes the clinical pathway, resource distribution, or performance evaluation.

Across these types, what ultimately differs is the prevailing care habitus: the routinised way in which care is enacted in everyday practice, shaped by institutional training, classificatory systems, protocols, and evaluation regimes (10). In non-reflexive healthcare cultures, these routines remain stabilised and taken for granted. In adaptive cultures, they are partially unsettled without being fundamentally reconfigured. Transformative healthcare cultures render these routines objects of collective reflection and institutional revision. What changes is the system’s ability to revise its own organising logic.

Returning to the migrant case introduced above helps clarify the analytical value of this framework. The same situation is configured differently depending on whether the prevailing healthcare culture is non-reflexive, adaptive, or transformative.

- In non-reflexive healthcare cultures, classification recodes structural exposure as individual pathology (e.g., “depression,” “non-compliance”), with no recording of racism, labour exploitation, or housing precarity. Knowledge validation centres on clinical assessment and adherence indicators, while the patient’s account of structural stressors has no decision-making weight. Criteria of efficiency prioritise rapid consultation, symptom control, and compliance. The result is a standardised pathway focused on pharmacological treatment and behavioural advice, with no modification based on structural conditions.
- In adaptive healthcare cultures, classification retains the clinical diagnosis while adding partial documentation of social determinants (e.g., unstable employment, housing insecurity). Knowledge validation incorporates elements of the patient’s narrative through structured histories or screening tools, with limited influence on decisions. Criteria of efficiency expand to include coordination and referral metrics. The pathway may include referral to social or community services, yet structural exposure remains weakly connected to decision authority and resource allocation.
- In transformative healthcare cultures, classification renders structural exposure systematically visible and decision-relevant. Knowledge validation integrates clinical assessment with the patient’s account of racism, labour conditions, and housing precarity as constitutive of the case. Criteria of efficiency incorporate equity-oriented outcomes, including reduction of structural vulnerability and improved capacity for participation. The pathway is reconfigured through coordinated clinical and social action, and structural exposure directly shapes diagnosis, intervention, and evaluation.

These contrasts reflect how variations in the configuration of the same mechanisms generate distinct care pathways and institutional responses. Table 1 summarises these differences.

**Table 1:** Configurations of healthcare cultures across key mechanisms.

Mechanism	Distinguishing criterion	Non-reflexive	Adaptive	Transformative
Classification	Is structural exposure recorded and decision-relevant?	Individualised diagnostic categories. Structural exposure absent or recoded as individual deficit	Diagnostic categories expanded with partial recording of social determinants. Weak linkage to decisions	Structural exposure systematically recorded and used to define cases and guide decisions
Knowledge validation	Does experiential knowledge carry decision authority?	Clinical and managerial evidence dominate. Experiential accounts marginal	Selective incorporation of patient narratives through structured tools. Limited decision weight	Experiential and community knowledge integrated into clinical reasoning and organisational deliberation
Criteria of efficiency	Are equity outcomes embedded in institutional evaluation?	Throughput, adherence, and risk control guide action. Equity external to evaluation	Mixed metrics combining clinical targets with coordination and referral indicators	Equity-oriented outcomes embedded in evaluation, including reduction of structural vulnerability and improved participation

Ideal-typical configurations based on the organisation of classification, knowledge validation, and criteria of efficiency. Empirical cases may combine elements across types.

These configurations are not merely ideal-typical. They can be empirically examined in routine clinical and organisational settings by tracing how classification, knowledge validation, and criteria of efficiency combine to produce specific care pathways and institutional responses. Table 2 presents a set of operational indicators, organised by mechanism and level of analysis, that can support this comparative work.

**Table 2:** Operational indicators for evaluating healthcare cultures across mechanisms and levels.

Mechanism	Level	Indicator	Data source	Diagnostic threshold + action
Classification	Micro	Structural determinants recorded in clinical records	Clinical records audit	Threshold: housing, racism, or labour precarity present as coded clinical categories. Action: modified care pathway triggered and documented.
Classification	Meso	Structural determinants embedded in institutional intake and triage criteria	Organisational audit	Threshold: structural exposure categories integrated into triage, eligibility, or intake protocols. Action: referral to interdisciplinary team initiated at point of triage.
Classification	Macro	National health information policies require structural determinants as operative categories in clinical systems	Policy and regulatory review	Threshold: structural determinants included as mandatory fields in national clinical information frameworks. Action: compliance monitored through national audit cycle.
Knowledge validation	Micro	Patient/community accounts included in clinical decision-making	Case review documentation	Threshold: narrative evidence formally registered in clinical notes. Action: documented influence on diagnosis, referral, or treatment plan.
Knowledge validation	Meso	Experiential knowledge formally recognised in organisational deliberation	Case conference records	Threshold: patient or community narratives present in case conference records. Action: narrative input traceable to change in case management or resource allocation.
Knowledge validation	Macro	Community representatives formally participate in system-level performance review or service design	Governance records	Threshold: community participation embedded in governance structures. Action: community input documented as influencing performance review outcomes or service redesign decisions.
Criteria of efficiency	Micro	Structural exposure modifies individual care pathway	Clinical audit	Threshold: structural exposure recorded. Action: change in diagnosis, referral, or resource allocation traceable to that exposure.
Criteria of efficiency	Meso	Equity-oriented outcomes incorporated in organisational performance regimes	Performance dashboard	Threshold: reduction of structural vulnerability included as auditable institutional target. Action: performance review triggers corrective protocol when target is not met.
Criteria of efficiency	Macro	Equity-oriented outcomes rewarded at system level	System evaluation framework	Threshold: equity-oriented outcomes present alongside throughput in national evaluation frameworks. Action: systems failing equity targets subject to governance review and resource reallocation.

Indicators are organised by the three analytical mechanisms and three levels of analysis introduced in the framework. The diagnostic threshold specifies the minimum observable condition for each indicator; the action specifies the institutional response that should follow. To illustrate: in a primary care centre, a micro-level Classification indicator would be met when a patient’s housing insecurity is coded in the electronic record as a clinically operative category, and the action triggered would be an automatic referral to a social care coordinator and a modified follow-up pathway. Data sources and thresholds are indicative and should be adapted to specific institutional and national contexts. Empirical cases may require combinations of indicators across levels.

Methodologically, this framework can be operationalised through a configurational analysis of routine clinical, organisational, and policy practices. Such analysis combines ethnographic observation of everyday care with institutional document analysis, tracing how classification, knowledge validation, and criteria of efficiency combine across micro, meso, and macro levels to produce specific care pathways and institutional responses. The typology and indicators presented in Tables 1, 2 provide the analytical scaffolding for this comparative work, allowing healthcare cultures to be identified, contrasted, and translated into evaluative and governance tools within public health systems.

## Discussion: implications for public health evaluation and governance

Much of the current equity gap can therefore be understood as a divide between knowing equity and doing equity. Health systems increasingly demonstrate awareness that equity matters, as reflected in policy frameworks, training programmes, and strategic commitments. What remains far less developed is the capacity to do equity in routine practice, that is, to enact it consistently through classifications, decisions, and institutional routines. Closing this gap requires institutional mechanisms that translate declared values into the classificatory systems and performance criteria of everyday practice.

Reframing culture as constitutive rather than contextual clarifies what current research and policy have left unaddressed: the absence of a shared institutional architecture capable of linking analysis, organisational design, and evaluation across system levels. Without this architecture, equity-oriented interventions are selectively absorbed without reconfiguring the cultural machinery through which healthcare is organised. The implications for evaluation, data systems, and governance are direct.

### Implications for evaluation

Health systems must move beyond downstream indicators (outcomes, coverage, or access) that privilege activity, efficiency, and compliance. Volumes of care, waiting times, throughput, adherence to protocols, and selected clinical outcomes remain indispensable for system management, yet they rarely assess whether structural vulnerability changes clinical pathways, resource allocation, or performance assessment. Evaluation frameworks must therefore be redesigned to make this visible. This requires a shift toward transformative healthcare cultures, one that does not imply abandoning standardisation or measurement, but demands a revision of what is standardised and measured. Systems such as NHS England’s Core20PLUS5 framework (25) or the CMS Framework for Health Equity (26) demonstrate that equity-oriented metrics can be institutionally embedded, yet they remain partial without reconfiguring the underlying cultural mechanisms that govern classification and knowledge validation.

Evaluation can be operationalised through a minimal set of indicators aligned with the three cultural mechanisms across micro, meso, and macro levels of the health system (see Table 2). The underlying principle is consequentiality: indicators are only meaningful when recorded exposures have traceable effects on clinical pathways, resource allocation, and performance evaluation. Indicators shape institutional priorities by defining what becomes visible, actionable, and accountable in everyday practice. What remains unevaluated tends to become institutionally invisible. Evaluation regimes therefore actively participate in the formation of healthcare cultures, not merely in their measurement.

### Implications for data systems

To move from diagnosis to evaluation, healthcare cultures must be rendered auditable through organisational signals embedded in routine infrastructures. Social determinants must be institutionally actionable within clinical and managerial systems. Electronic health records should allow structural exposures such as housing insecurity, racism, or labour precarity to be coded as clinically operative categories (27). This requires not only technical capacity but institutional mandate: coding fields must be linked to care pathways, not left as optional annotations. When recorded, these exposures should trigger modified care pathways, interdisciplinary referral, and resource reallocation. Coding without consequence does not alter care; auditability requires that recorded exposures have traceable effects on decisions and resource flows.

### Implications for governance

Case conferences and audit bodies should formally recognise narrative, community, and experiential accounts as valid forms of evidence alongside biomedical markers. Performance regimes should reward continuity, relational work, and reduction of structural exposure in addition to biomedical targets. Equity audit mechanisms, such as those developed by the NHS Race and Health Observatory (28) or the WHO equity-focused evaluation framework (29), offer practical models for institutionalising this revision at both national and system level. When inequities persist, health systems must subject organisational routines to institutional review and revision. This includes linking evaluation findings to decision authority, so that identified inequities prompt changes in protocols, resource allocation, and accountability structures. These signals establish a threshold test: a healthcare culture is transformative when structural determinants durably reshape classificatory systems, evidentiary hierarchies, and performance criteria.

## Conclusion

This article has argued that equity in healthcare is not primarily a problem of knowledge or intention, but of institutional organisation. A healthcare cultures perspective shifts attention upstream, from outcomes alone to the cultural processes through which problems are categorised, knowledge is validated, and efficiency is defined. The framework introduced here, built around three analytical mechanisms and a typology of non-reflexive, adaptive, and transformative healthcare cultures, offers tools that are both analytically precise and practically applicable across diverse health system contexts. This perspective reframes health as a socially produced capacity (30) and requires institutional arrangements that make structural conditions visible within decision-making systems. Under these conditions, equity becomes a property of how care is organised, governed, and evaluated in routine practice. Future research should examine how emerging technologies, including artificial intelligence in clinical decision-making, interact with these cultural mechanisms, amplifying or transforming classificatory hierarchies depending on the institutional cultures in which they are embedded (31). In the absence of such structural revision, inequities will persist as routine effects of everyday care.

## Data Availability

The original contributions presented in the study are included in the article/supplementary material, further inquiries can be directed to the corresponding author/s.

## References

[1] Solar O Irwin A. A Conceptual Framework for Action on the Social Determinants of Health. Geneva: World Health Organization (2010).

[2] Kirmayer LJ. Rethinking cultural competence. Transcult Psychiatry. (2012) 49:149–64. doi: 10.1177/1363461512444673 22508634

[3] Metzl JM Hansen H. Structural competency: theorizing a new medical engagement with stigma and inequality. Soc Sci Med. (2014) 103:126–33. doi: 10.1016/j.socscimed.2013.06.032, 24507917 PMC4269606

[4] Hansen H Metzl JM. New medicine for the US health care system: training physicians for structural interventions. Acad Med. (2017) 92:279–81. doi: 10.1097/ACM.0000000000001542, 28079725 PMC5540156

[5] Farmer P Nizeye B Stulac S Keshavjee S. Structural violence and clinical medicine. PLoS Med. (2006) 3:e449. doi: 10.1371/journal.pmed.0030449, 17076568 PMC1621099

[6] World Health Organization. Framework on Integrated, People-Centred Health Services. Geneva: WHO (2016).

[7] Cornwall A Jewkes R. What is participatory research? Soc Sci Med. (1995) 41:1667–76. doi: 10.1016/0277-9536(95)00127-S 8746866

[8] Greenhalgh T Papoutsi C. Studying complexity in health services research. BMC Med. (2018) 16:95. doi: 10.1186/s12916-018-1089-4, 29921272 PMC6009054

[9] Kleinman A. Patients and Healers in the Context of Culture. Berkeley: University of California Press (1980).

[10] Bourdieu P. Le sens pratique. Paris: Éditions de Minuit (1980).

[11] Good BJ. Medicine, Rationality, and Experience. Cambridge: Cambridge University Press (1994).

[12] Lock M Nguyen VK. An Anthropology of Biomedicine. Chichester: Wiley-Blackwell (2010).

[13] Napier AD Ancarno C Butler B Calabrese J Chater A Chatterjee H . Culture and health. Lancet. (2014) 384:1607–39. doi: 10.1016/S0140-6736(14)61603-2 25443490

[14] Scott T Mannion R Davies HTO Marshall MN. The quantitative measurement of organisational culture in health care. Health Serv Res. (2003) 38:923–45. doi: 10.1111/1475-6773.00154 12822919 PMC1360923

[15] Halligan M Zecevic A. Safety culture in healthcare. BMJ Qual Saf. (2011) 20:338–43. doi: 10.1136/bmjqs.2010.040964, 21303770

[16] Mannion R Davies HTO. Understanding organisational culture for healthcare quality improvement. BMJ. (2018) 363:k4907. doi: 10.1136/bmj.k4907 30487286 PMC6260242

[17] Power M. The Audit Society: Rituals of Verification. Oxford: Oxford University Press (1997).

[18] Tronto JC. Moral Boundaries: A Political Argument for An Ethic of Care. New York: Routledge (1993).

[19] Fraser N. Contradictions of capital and care. New Left Rev. (2016) 2:99–117. doi: 10.64590/nt2

[20] The Care Collective. The Care Manifesto: the Politics of Interdependence. London: Verso (2020).

[21] Scott WR. Institutions and Organizations. Thousand Oaks: Sage (2001).

[22] Martinez-Hernaez A Bekele D Sabariego C . The structural and intercultural competence for epidemiological studies (SICES) guidelines: a 22-item checklist. BMJ Glob Health. (2021) 6:e005237. doi: 10.1136/bmjgh-2021-005237, 33853845 PMC8728389

[23] Petersen EE Davis NL Goodman D . Racial/ethnic disparities in pregnancy-related deaths—United States, 2007–2016. MMWR Morb Mortal Wkly Rep. (2019) 68:762–5. doi: 10.15585/mmwr.mm6835a3, 31487273 PMC6730892

[24] Dayo E Christy K Habte R. Health in colour: black women, racism, and maternal health. Lancet Reg Health Am. (2023) 17:100408. doi: 10.1016/j.lana.2022.100408 36531129 PMC9735204

[25] NHS England. Core20PLUS5: An Approach to Reducing Healthcare Inequalities. London: NHS England (2021).

[26] Centers for Medicare and Medicaid Services. CMS Framework for Health Equity 2022–2032. Baltimore: CMS (2022).

[27] World Health Organization. Strategy for Optimizing national Routine Health Information Systems: Strengthening Routine Health Information Systems to Deliver Primary Health Care and Universal Health Coverage. Geneva: World Health Organization (2023).

[28] NHS Race and Health Observatory. Strategy 2025–2027: Driving race Equity in Health and care. London: NHS Confederation (2025).

[29] World Health Organization. Guidance Note on Integrating Health Equity, Gender Equality, Disability Inclusion and Human Rights in WHO Evaluations. Geneva: World Health Organization (2023).

[30] Bodini C Quaranta I. "COVID-19 in Italy: a new culture of healthcare for future preparedness". In: Manderson L Burke NJ Wahlberg A, editors. Viral Loads: Anthropologies of Urgency in the Time of COVID-19. London: UCL Press (2021). p. 443–55.

[31] Dankwa-Mullan I. Health equity and ethical considerations in using artificial intelligence in public health and medicine. Prev Chronic Dis. (2024) 21:240245. doi: 10.5888/pcd21.240245, 39173183 PMC11364282

